# Emerging infectious diseases--Southeast Asia.

**DOI:** 10.3201/eid0402.980201

**Published:** 1998

**Authors:** S K Lam

**Affiliations:** Department of Medical Microbiology, Faculty of Medicine, University of Malaya, Kuala Lumpur, Malaysia. lamsk@medicine.med.edu.my


### International Editors

### Emerging Infectious Diseases—Southeast Asia

**Sai Kit Lam**
University of Malaya, Kuala Lumpur, Malaysia

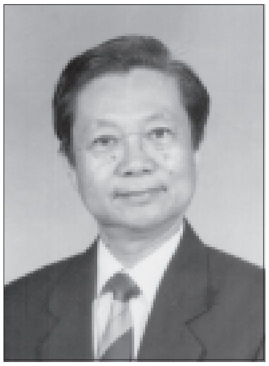

Dr. Lam is professor and head, Department of Medical Microbiology, Faculty of Medicine, University of Malaya. He serves on the editorial board of several medical journals and is the director of the WHO National Influenza Center, the WHO National Center for Rapid Viral Diagnosis, and the WHO Collaborating Center for Arbovirus Reference and Research for Dengue and Dengue Hemorrhagic Fever.

The recent emergence of a new strain (H5N1) of influenza A virus, the so-called avian or bird flu, in Hong Kong underlines the importance of the Southeast Asia region as an epicenter not only for influenza A viruses but also for other microbial agents. In late 1992, *Vibrio cholerae* O139 appeared on the Indian subcontinent; and within a few months, it had spread to China, Nepal, Pakistan, Malaysia, and as far as Russia.

The World Health Organization (WHO) has reported that infectious diseases account for more than 17 million deaths per year worldwide and that at least 30 new infectious diseases have emerged within the last 2 decades. Up to half of the 5.8 billion people on earth are at risk for many endemic diseases, with the most overpopulated and economically depressed countries in Southeast Asia at highest risk. Although vaccines and antibiotics are available for many diseases, in 1995 alone, respiratory infections such as pneumonia killed 4.4 million people, 4 million of them children. Diarrheal diseases, including cholera, typhoid, and dysentery killed 3.1 million, most of them children. Tuberculosis (TB) killed almost 3.1 million, malaria 2.1 million, hepatitis B more than 1.1 million, and measles more than 1 million.

Address for correspondence: S. K. Lam, Department of Medical Microbiology, Faculty of Medicine, University of Malaya, Kuala Lumpur, Malaysia; fax: 603-758-2801; e-mail: lamsk@medicine.med.um.edu.my.

Faced with the reality of infectious diseases on a daily basis, Southeast Asian nations have not been able to give emerging disease surveillance the priority status it deserves. Much of the surveillance in the region is centered in a few well-established laboratories where there is adequate expertise, sufficient funding, and personal interest. Because of influenza A, it is widely believed that Asia is the epicenter of new influenza strains; the emergence of the avian influenza strain in Hong Kong supports this contention. As a result of the activities of the Pacific Basin Respiratory Virus Research Group, an excellent surveillance program for respiratory viral infections is in place. If this small cluster of avian flu had occurred elsewhere, it would most likely have been missed. All the 110 WHO Influenza Centers, particularly those in Southeast Asia, have been alerted and are frequently updated about the Hong Kong outbreak. Diagnostic reagents for the identification of the H5N1 strain will be made available to these centers as part of the surveillance program.

### Enteroviruses

Enteroviruses are frequently associated with the hot and humid climate of tropical countries in Southeast Asia. The viruses are responsible mainly for inapparent and mild infection, but occasionally they can give rise to infection of the central nervous system, resulting in aseptic meningitis and (rarely) paralysis; polioviruses are a good example. Hand, foot, and mouth disease, caused by enteroviruses, such as coxsackie A16 and enterovirus 71, is common in many countries in the region. In a recent outbreak of hand, foot, and mouth disease in Malaysia, a new clinical entity of encephalomyelitis emerged, which resulted in several deaths among children under 5 years of age. Four such deaths were investigated thoroughly in the University Hospital in Kuala Lumpur. All four children were seen in the Emergency Department in a state of respiratory and cardiovascular instability with a history of fever (3 to 5 days) associated with reduced oral intake. One child had flaccid paralysis with hyporeflexia of the lower limbs. A constant feature in these patients was the very rapid vascular changes, followed rapidly by cardiac decompensation. Pulmonary edema followed, suggesting a concomitant increase in pulmonary vascular permeability. Consent for postmortem was obtained with some difficulty because of religious and cultural reasons. The midbrain, pons, medulla, and spinal cord were extensively damaged, with the worst damage at the level of the medulla. The cerebrum and myocardium were relatively normal. Enterovirus 71 was isolated from the medulla and spinal cord of three children and from the cerebrum of the other child, where no brain stem material was available. Other tissue sites including the cerebrospinal fluid did not yield virus. The need for postmortem examinations in investigating fatal emerging diseases must be addressed in areas where such examinations are not possible for cultural reasons.

### Arboviruses

Arthropod-borne viruses or arboviruses are yet another group of viruses synonymous with countries in the region and cause considerable sickness and death. Some of these viruses, e.g., Murray Valley encephalitis virus in Australia, have restricted geographic distribution but may have the ability to transcend geographical barriers, as in the emergence of Japanese encephalitis virus in the Torres Strait of northern Australia and in Papua New Guinea.

### Dengue

Dengue is by far the most important arbovirus infection in Southeast Asia. According to WHO, dengue has been reported in over 100 countries worldwide and poses a threat to approximately 2 billion people. At the 46th World Health Assembly held in Geneva in May 1993, a resolution was passed to make the prevention and control of dengue a priority.

Dengue is an acute viral infection characterized by abrupt onset of fever, severe headache, pain behind the eyes, muscle and joint pains, and rash. The hemorrhagic form of dengue fever, dengue hemorrhagic fever (DHF), was recognized as a new disease in the Philippines in 1953 and has been seen in India, Malaysia, Singapore, Indonesia, Vietnam, Cambodia, and Sri Lanka. During 1956 to 1992, 1,335,049 cases of DHF (13,723 deaths) were recorded in Vietnam alone. The rise of dengue in tropical and subtropical areas of the world is explained by factors such as rapid population growth, expanding urbanization, inadequate municipal water supplies, and difficulties in refuse disposal. These lead to an abundance of new breeding sites for the mosquito vectors, while human migration patterns disperse vectors and viruses into new areas.

The resurgence of DHF in several countries where only dengue fever had been reported has become worrisome. Sri Lanka reported its first outbreak in 1989, and India and China also faced the same dilemma. DHF/dengue shock syndrome has also been reported for the first time in New Caledonia and Tahiti. The large outbreak in Cuba in 1981 showed how the disease has spread to the Americas and other countries such as Venezuela and Brazil. In Malaysia, where the disease is endemic, encephalitis cases associated with dengue viruses have been documented, and vertical transmission has also been reported in the last two years. Other unusual manifestations observed rarely include acute renal failure and hemolytic uremic syndrome. The emergence of such unusual clinical manifestations should also be monitored and documented in other dengue-endemic countries. Despite intensive efforts by the countries in the region to control the vectors, the disease is still on the rise. A tetravalent live attenuated vaccine developed at Mahidol University, Thailand, is being field-tested, and the preliminary results are promising.

### Disease Spread

Rapid transportation has been blamed for the spread of diseases, and this is especially so in countries in the region. Tourism is an important industry in Southeast Asia, and a series of conferences on travel medicine has highlighted the problems, particularly in infectious diseases. Another vast movement of people between countries in the region is that of migrant workers. In Malaysia, an estimated 1.7 million workers are migrant workers, of which only 1.2 million came in legally. Most of the workers are from Indonesia, Myanmar, Philippines, and Pakistan, and they work in the agricultural sector as well as in construction and domestic services. Those who come in legally undergo a medical examination that includes screening for some infectious diseases. Diseases associated with migrant workers are chloramphenicol-resistant typhoid, multidrug-resistant TB, leprosy, malaria, HIV/AIDS, and filariasis. In 1993, the first case of kala-azar caused by *Leishmania donovani* was reported in a Bangladeshi migrant worker in Malaysia, and since then, four other cases have been identified, all of them in Bangladeshi workers. This disease had not been present in Malaysia.

In 1997, the theme “Emerging Infectious Diseases: Global Alert—Global Response” was chosen by WHO to celebrate World Health Day. This year, as WHO celebrates its 50th anniversary, the region is still plagued with major public health problems threatening the lives of millions of people in the region. These problems underline the need to strengthen epidemiologic surveillance and concentrate public health activities in Southeast Asia, the epicenter of many emerging diseases.

